# The Influence of Deep Hypothermic Circulatory Arrest on the Incidence of Postoperative Acute Kidney Injury After Pulmonary Endarterectomy

**DOI:** 10.3390/jcm15207931

**Published:** 2026-10-14

**Authors:** Dina Zdravković, Nikolaos Lafioniatis, Paraskevi Tsimpoura, René M’Pembele, Sebastian Roth, Eckhard Mayer, Christoph B. Wiedenroth, Stefan Guth, Ragnar Huhn, Carolin Torregroza

**Affiliations:** 1 Department of Anesthesiology, Kerckhoff-Clinic, Campus of Justus-Liebig-University Giessen, 61231 Bad Nauheim, Germany; d.zdravkovic@kerckhoff-klinik.de (D.Z.); n.lafioniatis@kerckhoff-klinik.de (N.L.); p.tsimpoura@kerckhoff-klinik.de (P.T.); r.huhn-wientgen@kerckhoff-klinik.de (R.H.); c.torregroza@kerckhoff-klinik.de (C.T.); 2 Department of Anesthesiology, Medical Faculty and University Hospital Duesseldorf, Heinrich-Heine-University Duesseldorf, 40225 Düsseldorf, Germany; sebastian.roth@med.uni-duesseldorf.de; 3 Department of Thoracic Surgery, Kerckhoff-Clinic, Campus of Justus-Liebig-University Giessen, 61231 Bad Nauheim, Germany; e.mayer@kerckhoff-klinik.de (E.M.); c.wiedenroth@kerckhoff-klinik.de (C.B.W.); s.guth@kerckhoff-klinik.de (S.G.); 4 Department of Anesthesiology, Amsterdam University Medical Center (AUMC), Location AMC, 1105 AZ Amsterdam, The Netherlands

**Keywords:** chronic thromboembolic pulmonary hypertension, pulmonary endarterectomy, deep hypothermic circulatory arrest, acute kidney injury

## Abstract

**Background:** Pulmonary endarterectomy (PEA) in patients with chronic thromboembolic pulmonary hypertension (CTEPH) is performed using phases of deep hypothermic circulatory arrest (DHCA). DHCA has been discussed as a risk factor for cardiac surgery-associated acute kidney injury (AKI). The study analyzes the influence of the duration of DHCA on the incidence of postoperative AKI in patients undergoing PEA. **Methods**: This retrospective single-center cohort study included adult CTEPH patients who underwent PEA. The main exposure was DHCA at 18–20 °C core temperature. Primary endpoint was postoperative AKI defined by the KDIGO serum creatinine criteria. Secondary endpoints included severe postoperative AKI, major adverse cardiovascular events and mortality. Univariate and multivariate logistic regression models were performed for risk factor analysis. **Results**: Four hundred and eighty-three patients were included (mean age 61 ± 13 years). The incidence of postoperative AKI was 50%. Multivariate analysis showed no association between DHCA and AKI. Postoperative AKI was independently associated with chronic kidney disease (OR = 1.76, 95% CI = 1.05–2.95), age (OR = 1.02, 95% CI = 1.00–1.03), cardiopulmonary bypass (CPB) time (OR = 1.01, 95% CI = 1.01–1.02) and vasoactive-inotropic score (VIS) (OR = 1.05, 95% CI = 1.01–1.10). Patients with preoperative diuretics had a lower risk for postoperative AKI (OR = 0.44, 95% CI = 0.29–0.66). MACE were more frequent in patients with postoperative AKI (28.4% vs. 14.2%, *p* < 0.001), whereas 30-day and 1-year mortality did not differ. **Conclusions**: Results from this study indicate no influence of DHCA duration on occurrence of postoperative AKI. Risk factors identified for postoperative AKI were chronic kidney disease, age, CPB duration and VIS.

## 1. Introduction

Cardiac surgery-associated acute kidney injury (CS-AKI) is a common complication with an incidence of 20 to 80% [1,2,3,4] and is strongly associated with mortality and length of hospitalization [1]. In the presence of chronic disease (heart, lung, liver) the susceptibility is even higher and the patients are more likely to suffer from postoperative AKI [5].

Chronic thromboembolic pulmonary hypertension (CTEPH) is a rare but life-threatening disease affecting patients of all age groups [6,7,8]. Although less invasive methods to treat CTEPH are gaining importance, pulmonary endarterectomy (PEA) remains the gold standard for the treatment of operable cases [9]. During the procedure deep hypothermic circulatory arrest (DHCA) is essential to obtain a bloodless surgical field required for optimal visualization of bilateral endarterectomy, as extensive collateral flow might otherwise obscure the operative site. Deep hypothermia serves to prevent the organs from ischemic damage during these periods of circulatory arrest. Therefore, the patient’s body temperature is lowered to 18 to 20 °Celsius [10].

In cardiac surgery, deep hypothermia is a common tool used for neuroprotection when circulatory arrest is needed. However, over the last years DHCA has been discussed to increase risk for renal dysfunction after proximal aortic surgery. So far, studies have shown inconsistent results. Calafiore et al. [11] recently provided evidence that DHCA is more effective than moderately hypothermic circulatory arrest combined with antegrade cerebral perfusion when it comes to renal protection.

AKI is not only common in patients undergoing cardiac surgery, but also a frequent complication after PEA. Only few studies have been conducted regarding AKI in this patient population with an incidence of AKI ranging between 26 and 45% [12].

This study aimed to analyze a potential effect of DHCA duration and identify further risk factors for postoperative AKI in CTEPH patients after PEA.

## 2. Materials and Methods

This single-center retrospective cohort study was approved by the Ethics Committee of the Justus-Liebig-University Giessen, Germany (amendment reference number: 199/15). Data were collected from the clinic’s digital patients’ records and in-house laboratory values, entered into a dedicated study database and afterwards extracted for analysis.

All patients ≥ 18 years who underwent PEA at the Kerckhoff Clinic, Bad Nauheim, Germany from January 2018 to December 2021 were screened for inclusion. Patients with missing data concerning DHCA duration or creatinine values were excluded as well as patients without a CTEPH diagnosis or missing data regarding secondary endpoints.

The main exposure of this study was DHCA duration measured in minutes. DHCA was defined as core blood temperature of 18–20 °C. Temperature was monitored intraoperatively via nasopharyngeal and bladder probes as well as blood temperature measured by heart-lung machine. The data was extracted from intraoperative charts, filled in by the supervising perfusionist at the time of surgery. If more than one period of DHCA was performed, the durations of all arrests were summed up.

All patients underwent standardized operative technique for PEA surgery as described previously [10,13]. Briefly, extracorporeal circulation was established via median sternotomy and cannulation of ascending aorta and placement of two venous cannulas into the superior and inferior vena cava. DHCA was implemented by systemically cooling via the heart-lung machine and defined as core blood temperature of 18–20 °Celsius. Intervals of DHCA were usually limited to 20 min each as per the institutional approach. In technically challenging cases, several intervals of DHCA are needed for bilateral endarterectomy. Given the narrow clinical distribution of DHCA times in this cohort (capped at a maximum of 20 min per ischemic period), DHCA was modeled as a continuous linear variable (per minute).

The primary endpoint was early AKI as defined by the “kidney disease: improving global outcomes” (KDIGO)-criteria occurring within 3 days after PEA. Creatinine values were measured in mg/dl preoperatively during admission, which was considered as baseline, and routinely on days one, two and three after surgery. Measurements were performed at the in-house laboratory. As urine output was not documented systematically, AKI was diagnosed based solely on creatinine values. AKI stage 1 was defined as a rise in serum creatinine to 1.5–1.9 times baseline within 7 days or an absolute rise by ≥0.3 mg/dL within 48 h. Stage 2 was defined as a rise of ≥2–2.9 times baseline and stage 3 as a rise of ≥3 times baseline, an absolute serum creatinine ≥ 4 mg/dL, or the initiation of renal replacement therapy. Secondary endpoints were severe early AKIs defined as KDIGO stage ≥ 2, major adverse cardiovascular events (MACE), 30-day mortality and 1-year mortality. MACE included cardiovascular death (CVD), stroke, non-fatal myocardial infarction, new arrhythmia (including atrial fibrillation) and new congestive heart failure and was recorded during in-hospital stay following PEA. Mortality was ascertained through reports originating from the centers that initially referred the patients to the Kerckhoff Clinic.

Statistical analysis was performed using IBM SPSS^©^ software version 27 (Armonk, NY, USA). Patient characteristics with continuous variables were presented as mean ± standard deviation (SD) or median and interquartile range (IQR) depending on normal distribution. When presenting categorical variables, numbers (n) with corresponding percentages (%) in brackets were used. To identify risk factors for primary and secondary endpoints, univariate and multivariate logistic regression analysis was performed. Additionally, a sensitivity analysis was conducted by restricting multivariate logistic regression to preoperative variables only. In line with the “events per variable” (EPV) recommendation, one variable per 10 events was included in the multivariate analysis. As data on AKI after PEA is scarce, covariates as pre-existing and perioperative risk factors were selected from studies investigating CS-AKI with DHCA [14,15,16,17]. As a result, the following covariates were included in the multivariate regression model: age (years), BMI, arterial hypertension, chronic kidney injury (defined by KDIGO guidelines as estimated GFR < 60 L/min/1.73 m^2^ at time of admission), preoperative treatment with diuretics, duration of DHCA (min), duration of surgery (min), duration of CPB (min), perioperative administration of fresh frozen plasma, and vasoactive-inotropic score. Perioperative FFP transfusions were entered into the logistic regression models as continuous variables (number of units transfused), whereas baseline characteristics in Table 1 were presented as categorical (any transfusion vs. no transfusion) where appropriate. To preserve statistical power due to sample size, propensity score matching was not performed. Baseline characteristics that differed between both study groups were controlled by inclusion in univariate and multivariate logistic regression models. Multicollinearity among independent predictors was assessed using Variance Inflation Factors (VIF). *p* < 0.05 was considered statistically significant for all tests.

## 3. Results

### 3.1. Study Population and Patient Characteristics

**Figure 1:**
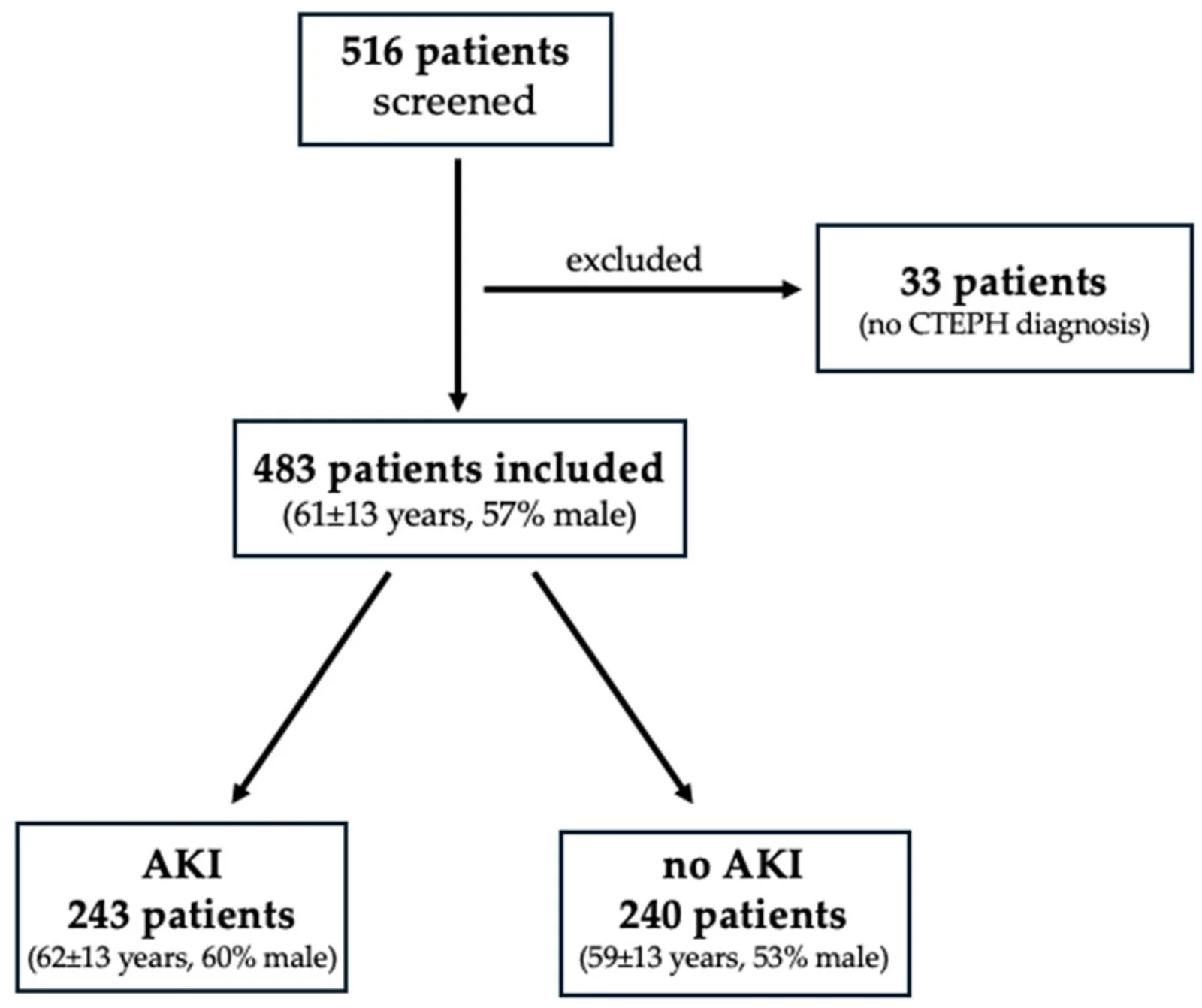
Study flow chart. Patient flow diagram of all patients undergoing PEA. AKI = Acute kidney injury (defined by KDIGO serum creatinine criteria).

A total of 516 patients underwent PEA surgery during the study period. Thirty-three patients without CTEPH diagnosis were excluded from analysis (Figure 1). As there was no missing data regarding primary and secondary endpoints, a total of 483 patients were included in the analysis. Two hundred seventy-three patients were male (56.5%), 210 female (43.5%) and the mean age was 61 ± 13 years (range 20–85 years). Detailed patient characteristics can be found in Table 1.

### 3.2. Primary Endpoints

#### 3.2.1. Postoperative AKI

The overall incidence of postoperative AKI was 50.3% (60.1% male, mean age 62 ± 13 years). Out of these, 212 were classified as AKI Stage I, 30 patients had AKI Stage II and one patient developed AKI Stage III. There was no difference in mean DHCA duration between patients with (39.2 ± 11 min) and without AKI (38.8 ± 9 min, *p* = 0.657). The univariate regression analysis showed no statistically significant association of DHCA duration and the incidence of postoperative AKI after PEA (OR 1.00, 95% CI: 0.99–1.02, *p* = 0.690).

Preoperative chronic kidney disease (OR = 1.76, 95% CI = 1.05–2.95, *p* = 0.031), age (OR = 1.02, 95% CI = 1.00–1.03, *p* = 0.042), CPB duration (OR = 1.01, 95% CI = 1.01–1.02, *p* < 0.001) as well as vasoactive-inotropic score (VIS) (OR = 1.05, 95% CI = 1.01–1.10, *p* = 0.010) were independently associated with early AKI in multivariate logistic regression analysis. Preoperative use of diuretics was inversely associated with postoperative AKI (OR = 0.44, 95% CI = 0.29–0.66, *p* < 0.001). All included variables exhibited a VIF under 2.5, indicating no critical collinearity.

To exclude potential confounding from early postoperative variables (e.g., VIS), a sensitivity analysis was performed by restricting the multivariate logistic regression model strictly to preoperative factors (shown in Table 2). In this model, advanced age (aOR 1.02, 95% CI = 1.00–1.03, *p* = 0.02), higher BMI (aOR 1.04, 95% CI = 1.00–1.07, *p* = 0.043) and preoperative diuretic therapy (aOR 0.53, 95% CI = 0.36–0.78, *p* = 0.001) remained independently associated with postoperative AKI. Preexisting CKD marginally missed statistical significance (aOR 1.58, 95% CI = 0.96–2.60, *p* = 0.071).

#### 3.2.2. Severe Postoperative AKI

The overall incidence of severe early AKI (stage ≥ 2) was 6.5% (61.3% male) with a mean age of 61 ± 16 years. There was no difference in DHCA duration between patients with (39.5 ± 11 min) and without severe AKI (38.9 ± 10 min).

The multivariate logistic regression showed an independent association for severe AKI and the perioperative administration of fresh frozen plasma (OR = 6.81, CI = 3.18–14.55, *p* < 0.001).

### 3.3. Secondary Endpoints

Overall mortality was low with a 30-day mortality rate of 1.7% and 1-year mortality of 2.5%. The logistic regression showed no association for DHCA duration and 30-day (OR = 0.93, 95% CI = 0.87–1.00, *p* = 0.056) or 1-year mortality (OR = 0.98, 95% CI = 0.92–1.04, *p* = 0.44).

A significantly higher occurrence of MACE was detected in patients with postoperative AKI (28.4% vs. 14.2%, *p* < 0.001). Duration of DHCA did not correlate with MACE in the logistic regression (OR = 0.98, 95% CI = 0.96–1.01, *p* = 0.147).

## 4. Discussion

Deep hypothermic circulatory arrest (DHCA) is the standard procedure during bilateral pulmonary endarterectomy (PEA) in patients suffering from chronic thromboembolic pulmonary hypertension (CTEPH) [10,13]. DHCA has been studied extensively in cardiothoracic surgery as neuroprotection, but potential implication on renal function remains inconclusive. The present study demonstrated high incidences of postoperative AKI, affecting 50% of patients following PEA. However, intraoperative DHCA was not independently associated with occurrence of either early or severe postoperative AKI. Instead chronic kidney disease and advanced age were shown as independent pre-existing risk factors, whereas prolonged cardiopulmonary bypass (CPB) duration and vasoactive-inotropic score (VIS) were shown as perioperative factors associated with postoperative AKI after PEA. In contrast to these findings, preoperative diuretic use was associated with lower odds of postoperative AKI. Lastly, patients who received perioperative fresh frozen plasma (FFP) had higher odds for severe early AKI in this study population.

Acute kidney injury after cardiac surgery is a frequent complication with studies reporting a wide range of incidence between 20 and 80% depending on the definition used [1,2,3,4]. For patients undergoing PEA surgery the incidence of postoperative AKI has been reported between 26% and 45% [12]. Our study showed an incidence of 50% for early postoperative AKI defined by KDIGO serum creatinine criteria. These findings are in line with a recently published study from Wang et al. reporting an incidence of 49% in postoperative AKI after PEA [12]. In contrast to our data, they observed higher in-hospital and 5-year mortality rates in patients with postoperative AKI. However, their cohort had a considerably higher overall mortality rate (30-day mortality: 3.7%; AKI stage ≥ 2: 12.5%) compared to our study possibly explaining the difference in outcome data. Further, they detected BMI, age, low preoperative hemoglobin, creatinine levels and low ejection fraction as risk factors for AKI. A hemoglobin level of 12.8 g/dL was presented as the threshold to distinguish occurrence of postoperative AKI [12]. Patients included in our study had average preoperative hemoglobin levels above this threshold, which could explain why hemoglobin levels were not associated with postoperative AKI in our study population.

More risk factors for AKI after PEA were identified by Zhang et al. in a retrospective single-center cohort study [14]. Next to preoperative hemoglobin levels, they showed an independent association between AKI and preoperative platelet count as well as duration of DHCA. Comparing the preoperative platelet count reported by Zhang et al. patients in the current study had overall higher reported values, especially when comparing patients with AKI in both studies [14]. These results emphasize the importance of preoperative values in patients undergoing PEA surgery and should be investigated in more detail in further studies.

Our statistical analysis did not show any independent association for DHCA and early or severe early AKI. These results are contrary to the findings of Zhang et al., even though DHCA duration and core temperature were comparable and similar risk factors were included in multivariate analysis in both studies [14]. The contradictory results could be explained by differences in patient characteristics (e.g., lower mean age in the study of Zhang et al.) and study size (n = 123 vs. n = 483) [14]. Besides the above-mentioned studies, data on AKI after PEA is scarce and thus it is unavoidable to also compare our findings to cardiac surgery patients with intraoperative DHCA. In this group, reported incidences of CS-AKI were similar to our findings (45–50%) [15,16]. Increased CPB duration, advanced age, elevated BMI and preoperative kidney injury among others have been identified as independent risk factors for CS-AKI [16]. These findings are in line with results on risk factors from our study. Of note, preoperative diuretic therapy was associated with lower odds of postoperative AKI in our cohort. This inverse association should be interpreted with caution: in a retrospective design, diuretic use is more likely to reflect the underlying clinical phenotype and volume status of the patient than a nephroprotective drug effect, and confounding by indication cannot be excluded. With regard to DHCA and the occurrence of postoperative AKI in cardiac surgery patients, study results are inconclusive. Englberger et al. found no independent association between DHCA duration and CS-AKI in 851 patients undergoing thoracic aortic surgery [17]. While target core temperature for DHCA was comparable to our study, they showed a lower rate of postoperative AKI (overall 17.7%). However, there are several studies that have identified DHCA as an independent risk factor of CS-AKI [18,19]. Duration of DHCA seems to play a pivotal role in occurrence of renal failure after thoracic arch surgery. A meta-analysis including over 4000 patients compared the effect of DHCA versus moderate hypothermic circulatory arrest (MHCA) on postoperative AKI in patients undergoing arch surgery. Results showed a reduction in incidence of AKI in the MHCA group compared to the DHCA group; however, when circulatory arrest time exceeded 30 min no difference was detected between both groups [20]. The impact of temperature during hypothermic circulatory arrest (HCA) on postoperative AKI was further investigated by Vekstein et al. in patients undergoing aortic surgery [21]. Multivariate analysis showed no association between degree of hypothermia and AKI with a median systemic circulatory arrest time of 17 min. The duration of DHCA is particularly important when looking at the occurrence of AKI in PEA patients. At the Kerckhoff Clinic as well as other specialized centers, DHCA is limited to episodes of 20 min or less when performing PEA with intermittent phases of reperfusion. Cumulative DHCA duration may not be physiologically equivalent to a single continuous period of circulatory arrest (as often used in cardiac surgery). Intermittent reperfusion between ischemic periods may provide a preconditioning effect, potentially mitigating ischemic renal injury.

Lastly, we could show an independent association between severe early postoperative AKI and the perioperative administration of FFP. None of the above-mentioned studies investigating risk factors for AKI after PEA formed a subgroup of patients suffering from AKI stage ≥ 2 [12,14,22]. Thus, there is no study population of PEA patients to compare our findings to. Kim et al. published a retrospective study in 2015 using the data of patients undergoing surgery on the thoracic aorta with CPB. Propensity score matching was used to compare 183 AKI-patients with non-AKI patients in their study population [23]. Multivariate regression detected an independent association for AKI and the transfusion of FFP. Although they did not differ between AKI and severe AKI, their results support our finding. Similar results were recently shown by Vlasov et al. in cardiac surgery patients [24]. Administration of FFP might be a surrogate parameter for massive intraoperative bleeding. Therefore, they performed a subgroup analysis to investigate whether intraoperative bleeding is an independent risk factor for CS-AKI. Their results indicate that hypotension and higher fluid balance (by administration of FFP) are the main risk factors and should be prevented during surgery. Our study did not focus on perioperative hemodynamics or fluid balance; thus, data on perioperative FFP administration and postoperative AKI is merely descriptive and no conclusion can be drawn about the cause of association. Multicenter studies are needed to investigate these risk factors in a larger study population.

### Strengths and Limitations

The retrospective single-center cohort study design potentially limits the external applicability of the results, and residual confounding cannot be excluded. Nevertheless, compared with other studies on postoperative AKI after PEA, the number of patients included is comparatively large. AKI was classified using the serum creatinine criteria only, as urine output was not documented systematically; the incidence reported here may therefore differ from studies applying the full KDIGO definition. In addition, the observation period was restricted to the first three postoperative days, so that later-onset AKI would not have been captured. While we did report preoperative diuretic therapy, other concomitant medication—in particular the use of nephrotoxic drugs—and perioperative contrast medium exposure were not included in this analysis.

Lastly, the low incidence of severe postoperative AKI but also the low mortality rates limit statistical analysis for these endpoints.

## 5. Conclusions

Postoperative AKI (defined by KDIGO serum creatinine criteria) occurred in half of all patients following PEA surgery; however, the rate of severe early AKI was only 6.5% in these patients. There was no statistically significant association of the duration of DHCA on postoperative AKI after PEA. Preoperative chronic kidney disease, advanced age, prolonged CPB duration and higher VIS were identified as independent risk factors for postoperative AKI, while preoperative use of diuretics was associated with lower odds of AKI in this cohort.

## Figures and Tables

**Table 1:** Patient, surgical and outcome characteristics.

Characteristics	Full Dataset (n = 483)	AKI (n = 243)	No AKI (n = 240)	*p*-Value
**Baseline characteristics**				
Age (years)	61 ± 13	62 ± 13	59 ± 13	0.008
BMI	29 ± 6	29.1 ± 5.6	28 ± 5.8	0.036
Sex (male)	273 (57%)	146 (60%)	127 (53%)	0.112
Preoperative Hemoglobin level (g/dL)	14.6 ± 1.7	14.6 ± 1.7	14.6 ± 1.8	0.863
Preoperative Thrombocytes (×10^3^/µL)	250 ± 94	251.8 ± 103.3	247.1 ± 83.6	0.584
Preoperative Creatinine level (mg/dL)	1.0 ± 0.5	1 ± 0.4	1.0 ± 0.6	0.907
NYHA ≥ 3	236 (49%)	111 (46%)	125 (52%)	0.325
**Comorbidities**				
Arterial hypertension	253 (52%)	143 (59%)	110 (46%)	0.004
Diabetes mellitus	100 (21%)	45 (19%)	55 (23%)	0.233
Chronic kidney disease	90 (19%)	54 (22%)	36 (15%)	0.044
HFrEF	5 (1%)	3 (1.2%)	2 (0.8%)	0.66
Preoperative diuretic therapy	266 (55%)	123 (51%)	143 (60%)	0.048
**Surgery characteristics**				
Duration of surgery (min)	411 ± 61	422 ± 64	401 ± 55	<0.001
Duration of CPB (min)	274 ± 42	283 ± 42	265 ± 40	<0.001
Duration of DHCA (min)	39 ± 10 39 (33–45)	39 ± 11 39 (33–46)	39 ± 9 39 (33–44)	0.657
Number of DHCA episodes	2 (2–3)	2 (2–3)	2 (2–3)	0.556
Patients with > 2 DHCA episodes	132 (27%)	67 (28%)	65 (27%)	0.904
Maximum postoperative VIS (within 24 h)	3.7 (1.4–7.4)	4.4 (2–8.8)	3.3 (1.2–6.3)	0.005
Perioperative PRBCs transfusion	187 (39%)	103 (42%)	84 (35%)	0.096
Perioperative FFP transfusion	70 (15%)	48 (20%)	22 (9%)	<0.001
**Outcome**				
MACE	103 (21%)	69 (28%)	34 (14%)	<0.001
ECMO	17 (4%)	11 (5%)	6 (3%)	0.198
ICU stay (days)	1 (1–2)	1 (1–2)	1 (1–2)	0.009
Duration of mechanical ventilation (hours)	14 (12–17)	14 (12–18)	14 (13–16)	0.007
30-day mortality	8 (1.7%)	5 (2%)	3 (1.3%)	0.483
1-year mortality	12 (2.5%)	9 (3.7%)	3 (1.3%)	0.083
CVVHD	15 (3%)	11 (5%)	4 (1.7%)	0.07
Reperfusion pulmonary edema	103 (21%)	57 (24%)	46 (19%)	0.25

Patient, surgical and outcome characteristics. AKI = Acute kidney injury (defined by KDIGO serum creatinine criteria); BMI = Body mass index; NYHA = New York Heart Association; HFrEF = Heart failure with reduced ejection fraction; CPB = Cardiopulmonary bypass; DHCA = Deep hypothermic circulatory arrest; VIS = Vasoactive-inotropic score; PRBCs = Packed red blood cells; FFP = Fresh frozen plasma; MACE = Major adverse cardiovascular events; ECMO = Extracorporeal membrane oxygenation; ICU = Intensive Care Unit; CVVHD = Continuous veno-venous hemodialysis.

**Table 2:** Univariate and multivariate logistic regression models.

	Univariate Analysis		Multivariate Analysis		Preoperative Sensitivity Model	
Variables	OR (95% CI)	*p*-Value	OR (95% CI)	*p*-Value	OR (95% CI)	*p*-Value
**Sex**	1.34 (0.93–1.92)	0.113				
**Age**	1.02 (1.01–1.03)	**0.009**	1.02 (1.00–1.03)	**0.042**	1.02 (1.00–1.03)	**0.020**
**BMI**	1.03 (1.00–1.07)	**0.037**	1.04 (0.99–1.08)	0.066	1.04 (1.01–1.07)	**0.043**
**Arterial hypertension**	1.69 (1.18–2.42)	**0.004**	1.44 (0.95–2.18)	0.087	1.48 (0.99–2.20)	0.055
**CKD**	1.61 (1.01–2.57)	**0.045**	1.76 (1.05–2.95)	**0.031**	1.58 (0.96–2.60)	0.071
**Preoperative Creatinine level (mg/dL)**	0.98 (0.67–1.44)	0.907				
**Preoperative Thrombocytes (×10^3^/µL)**	1.00 (1.00–1.00)	0.583				
**Preoperative Hemoglobin level (g/dL)**	0.99 (0.89–1.10)	0.862				
**Preoperative Diuretics**	0.70 (0.49–1.00)	**0.048**	0.44 (0.29–0.66)	**<0.001**	0.53 (0.36–0.78)	**0.001**
**Duration of DHCA (min)**	1.00 (0.99–1.02)	**0.690**	0.98 (0.96–1.00)	0.076		
**Duration of surgery (min)**	1.01 (1.00–1.01)	**<0.001**	1.00 (0.99–1.01)	0.447		
**Duration of CPB (min)**	1.01 (1.01–1.02)	**<0.001**	1.01 (1.01–1.02)	**<0.001**		
**Perioperative FFP transfusion (per unit)**	1.14 (1.04–1.26)	**0.008**	1.54 (0.84–2.83)	0.166		
**VIS (within 24 h)**	1.05 (1.01–1.08)	**0.007**	1.05 (1.01–1.10)	**0.010**		

Univariate and multivariate logistic regression models for postoperative acute kidney injury (defined by KDIGO serum creatinine criteria). BMI = Body mass index; CKD = Chronic kidney disease; CPB = Cardiopulmonary bypass; VIS = vasoactive-inotropic score; FFP = Fresh frozen plasma; OR = odds ratio; CI = confidence interval; OR for FFP represents the risk increment per single unit of FFP transfused.

## Data Availability

The data presented in this study are available on request from the corresponding author due to ethical restriction.

## Abbreviations

The following abbreviations are used in this manuscript:

AKI	Acute kidney injury
BMI	Body mass index
CI	Confidence interval
CPB	Cardiopulmonary bypass
CS-AKI	Cardiac surgery-associated acute kidney injury
CTEPH	Chronic thromboembolic pulmonary hypertension
CVD	Cardiovascular death
CVVHD	Continuous veno-venous hemodialysis
DHCA	Deep hypothermic circulatory arrest
ECMO	Extracorporeal membrane oxygenation
FFP	Fresh Frozen Plasma
HCA	Hypothermic circulatory arrest
HFrEF	Heart failure with reduced ejection fraction
IQR	Interquartile range
KDIGO	Kidney Disease: Improving Global Outcomes
MACE	Major adverse cardiac events
MHCA	Moderate hypothermic circulatory arrest
NYHA	New York Heart Association
OR	Odds ratio
PEA	Pulmonary endarterectomy
PRBC	Packed red blood cells
SD	Standard deviation
VIS	Vasoactive-inotropic score
